# Artificial Intelligence in Cardiac Treatment Decision-Making: An Evaluation of the Performance of ChatGPT Versus the Heart Team in Coronary Revascularization

**DOI:** 10.31083/RCM38705

**Published:** 2025-08-19

**Authors:** Serkan Mola, Alp Yıldırım, Enis Burak Gül

**Affiliations:** ^1^Cardiovascular Surgery Department, Ankara Bilkent City Hospital, 06800 Ankara, Turkey; ^2^Cardiovascular Surgery Department, Ankara Atatürk Sanatoryum Training and Research Hospital, 06290 Ankara, Turkey

**Keywords:** artificial intelligence, ChatGPT, coronary revascularization, heart team

## Abstract

**Background:**

This study aimed to investigate the performance of two versions of ChatGPT (o1 and 4o) in making decisions about coronary revascularization and to compare the recommendations of these versions with those of a multidisciplinary Heart Team. Moreover, the study aimed to assess whether the decisions generated by ChatGPT, based on the internal knowledge base of the system and clinical guidelines, align with expert recommendations in real-world coronary artery disease management. Given the increasing prevalence and processing capabilities of large language models, such as ChatGPT, this comparison offers insights into the potential applicability of these systems in complex clinical decision-making.

**Methods:**

We conducted a retrospective study at a single center, which included 128 patients who underwent coronary angiography between August and September 2024. The demographics, medical history, current medications, echocardiographic findings, and angiographic findings for each patient were provided to the two ChatGPT versions. The two models were then asked to choose one of three treatment options: coronary artery bypass grafting (CABG), percutaneous coronary intervention (PCI), or medical therapy, and to justify their choice. Performance was assessed using metrics such as accuracy, sensitivity, specificity, precision, F1 score, Cohen's kappa, and Shannon's entropy.

**Results:**

The Heart Team recommended CABG for 78.1% of the patients, PCI for 12.5%, and medical therapy for 9.4%. ChatGPT o1 demonstrated higher sensitivity in identifying patients who needed CABG (82%) but lower sensitivity for PCI (43.7%), whereas ChatGPT 4o performed better in recognizing PCI candidates (68.7%) but was less accurate for CABG cases (43%). Both models struggled to identify patients suitable for medical therapy, with no correct predictions in this category. Agreement with the Heart Team was low (Cohen's kappa: 0.17 for o1 and 0.03 for 4o). Notably, these errors were often attributed to the limited understanding of the model in a clinical context and the inability to analyze angiographic images directly.

**Conclusion:**

While ChatGPT-based artificial intelligence (AI) models show promise in assisting with cardiac care decisions, the current limitations of these models emphasize the need for further development. Incorporating imaging data and enhancing comprehension of clinical context is essential to improve the reliability of these AI models in real-world medical settings.

## 1. Introduction

Large language models (LLMs) have undergone rapid and significant evolution in recent years, demonstrating their capability to utilize “big data”, understand human-input prompts, generate context-aware responses, and even mimic expert-level decision-making. These models exhibit high linguistic fluency and adaptability, and have proven their potential in multiple fields [1, 2, 3]. In the medical field, LLMs have advanced to the point of passing standardized board examinations and providing diagnostic suggestions in complex clinical scenarios [4, 5]. One of the most widely recognized LLMs is ChatGPT, developed by OpenAI.

In the 2021 European Society of Cardiology and European Association for Cardio-Thoracic Surgery guidelines, the concept of a multidisciplinary team comprising cardiologists, cardiac surgeons, radiologists, and cardiac anesthesiologists was introduced under the term “Heart Team” [6]. This collaborative approach has been emphasized as pivotal in surgical decision-making in cardiac care [7]. Specifically, it is recommended that the Heart Team evaluate complex coronary artery revascularization and valvular pathologies [8]. However, in actual clinical practice, such multidisciplinary collaborations may not always be consistently implemented, and there are health centers where a formal Heart Team does not exist or function as outlined in the guidelines [9, 10].

Given ChatGPT’s ability to process large amounts of data and mimic expert-level reasoning within the limits of the data provided, we aimed to examine its potential contribution to Heart Team deliberations. In particular, we investigated how ChatGPT’s recommendations align with those made by specialized human teams of cardiologists, surgeons, and other professionals. In addition, we examined the factors that may explain any differences between these two decision-making processes.

## 2. Methods

This was a retrospective single-center study that included patients who underwent coronary angiography between August and September 2024 and were deemed to require Heart Team evaluation. In accordance with our study protocol, patients who underwent emergent coronary artery bypass grafting (CABG) or coronary angiography were not included in this cohort. In addition, any lesion that involved the left anterior descending (LAD) artery was invariably evaluated by the multidisciplinary Heart Team to ensure a comprehensive assessment guided by input from multiple specialties. A total of 128 consecutive patients were enrolled in the study. Information on the patients’ demographics, comorbidities, angiographic data (including coronary angiograms), and relevant clinical details were collected. In our multidisciplinary Heart Team, four distinct specialties are routinely represented: cardiology, cardiovascular surgery, anesthesiology, and radiology. During the decision-making phase, at least two cardiologists and two cardiovascular surgeons are required to participate. This composition ensures a balanced and comprehensive patient evaluation, as it integrates both medical and surgical perspectives. Heart Team discussions were conducted in accordance with the most recent literature and revascularization guidelines, supplemented by clinical expertise [8, 11]. Local ethics committee approval (Ethics No. TABED 1-24-539) was obtained before study commencement, and all the participants provided written informed consent for the use of their data.

To investigate the potential role of ChatGPT in decision-making, we used two models, ChatGPT 4o and ChatGPT o1. Data on each patient’s demographic information, comorbidities, current medications, echocardiographic findings, and coronary angiogram were fed into the ChatGPT models. We then issued the following prompt:

I am providing you with a patient’s demographic information, comorbidities, current medications, echocardiographic findings, and coronary angiography diagram. Based on these data and in light of the latest guidelines, I would like you to select one of the following three options and justify your choice in a single sentence:

(A) CABG is required.

(B) Percutaneous coronary intervention (PCI) is required.

(C) Medical therapy alone is sufficient.

Both ChatGPT 4o and ChatGPT o1 were presented with the same data and prompt, and their respective responses were recorded for subsequent analysis.

## 3. Statistical Analysis

We compared Heart Team decisions with the ChatGPT responses obtained from the 4o and o1 models. Agreement and performance were evaluated using accuracy, sensitivity, specificity, precision, F1 score, Shannon’s entropy, and Cohen’s kappa.

Categorical variables were compared using the chi-square or Fisher exact test, where appropriate, on the basis of expected cell counts. For continuous variables, normality was assessed using the Shapiro-Wilk test. Normally distributed variables were compared across the three treatment groups (CABG, PCI, and medical therapy) using a one-way analysis of variance. In cases where normality assumptions were not met, the Kruskal-Wallis *H* test was used as a nonparametric alternative to evaluate differences in medians. A *p* value < 0.05 was considered statistically significant for all analyses.

## 4. Performance Metrics

To evaluate the agreement between ChatGPT’s recommendations and the multidisciplinary Heart Team’s decisions, we calculated the following key classification performance metrics:

True Positives (TP): Cases correctly classified as requiring a given intervention.

True Negatives (TN): Cases correctly identified as not requiring the intervention.

False Positives (FP): Cases incorrectly classified as requiring the intervention.

False Negatives (FN): Cases incorrectly classified as not requiring the intervention.

Accuracy was defined as the percentage of correctly classified cases among all cases, computed as follows: Accuracy = (TP + TN)/(TP + TN + FP + FN).

Sensitivity measured the model’s ability to correctly identify positive cases and calculated as follows: Sensitivity (Recall) = TP/(TP + FN).

Specificity represented the percentage of correctly identified negative cases: Specificity = TN/(TN + FP).

Precision (positive predictive value [PPV]) was assessed as the percentage of true positives among all predicted positives: Precision = TP/(TP + FP).

F1 score is the harmonic mean of precision and recall: F1 = 2 × (Precision × Recall)/(Precision + Recall).

### 4.1 Shannon’s Entropy

*Definition:* Measure of the distribution (uncertainty) of predicted classes.

*Formula: H* = –∑ (*p*ᵢ × log₂(*p*ᵢ)), where *p*ᵢ represents the percentage of predictions in class *i*.

### 4.2 Cohen’s Kappa (κ)

*Definition:* Evaluate agreement between the model’s predictions and a reference standard, adjusted for chance.

*Formula: κ* = (*p*₀ – *p*ₑ)/(1 – *p*ₑ), where *p*₀ is the observed agreement, and *p*ₑ is the expected agreement under a random model.

These metrics were used to compare the diagnostic performances of the ChatGPT models and formats against expert clinical decisions.

In this study, the Python programming language was used to compute the confusion matrix by comparing the predictions of ChatGPT 4o and o1 with the Heart team’s results using scikit-learn (confusion_matrix function). The resulting matrices were visualized using Seaborn (heatmap function) and Matplotlib.

## 5. Results

In this study, 128 patients were included, with a mean (± SD) age of 63 ± 9 years. Of these patients, 23.4% were female (Table 1). Hypertension and diabetes were present in 45.3% and 46.1% of the cohort, respectively. The overall median ejection fraction (EF) was 55% (interquartile range, 47%–60%). The Heart Team recommended CABG for 100 of the 128 patients evaluated, PCI for 16 patients, and maximal medical therapy for the remaining 12 patients.

**Table 1.** **Patients’ demographic data**.

		All Patients (n = 128)	CABG (n = 100)	PCI (n = 16)	Medical Therapy (n = 12)	*p*
Age (mean ± SD)		63 ± 9	63 ± 8	62 ± 13	60 ± 9	0.480^a^
Female, n (%)		30 (23.4)	22 (22)	6 (37.5)	2 (16.7)	0.330^b^
Hypertension, n (%)		58 (45.3)	50 (50)	4 (25)	4 (33.3)	0.120^b^
Diabetes, n (%)		59 (46.1)	48 (48)	4 (25)	7 (58.3)	0.150^b^
Morbid obesity, n (%)		4 (3.1)	1 (1)	0 (0)	3 (25)	0.040^c^
COPD, n (%)		13 (10.2)	12 (12)	1 (6.3)	0 (0)	0.580^c^
Valve pathology, n (%)		10 (7.8)	8 (8)	0 (0)	2 (16.7)	0.250^c^
EF, median (IQR), (%)		55 (47–60)	55 (50–60)	60 (42–60)	55 (35–60)	0.590^d^
Number of diseased vessels, n (%)						
	One	10 (7.8)	6 (6)	3 (18.7)	1 (8.3)	<0.001^c^
	Two	4 (3.1)	1 (1)	1 (6.3)	2 (16.7)	
	Three	66 (51.6)	46 (46)	12 (75)	8 (12.1)	
	Four and more	48 (37.5)	47 (47)	0 (0)	1 (2.7)	

CABG, Coronary Artery Bypass Grafting; PCI, Percutaneous Coronary Intervention; COPD, Chronic Obstructive Pulmonary Disease; EF, Ejection Fraction. ^a^One-way analysis of variance. ^b^Chi-square test. ^c^Fisher exact test. ^d^Kruskal-Wallis *H* test.

The distribution of diseased vessels differed significantly (*p* = 0.001). Three or more extensively diseased vessels were observed in 93% of the CABG candidates, 75% of the PCI candidates, and 75% of those managed medically.

**Fig. 1.**
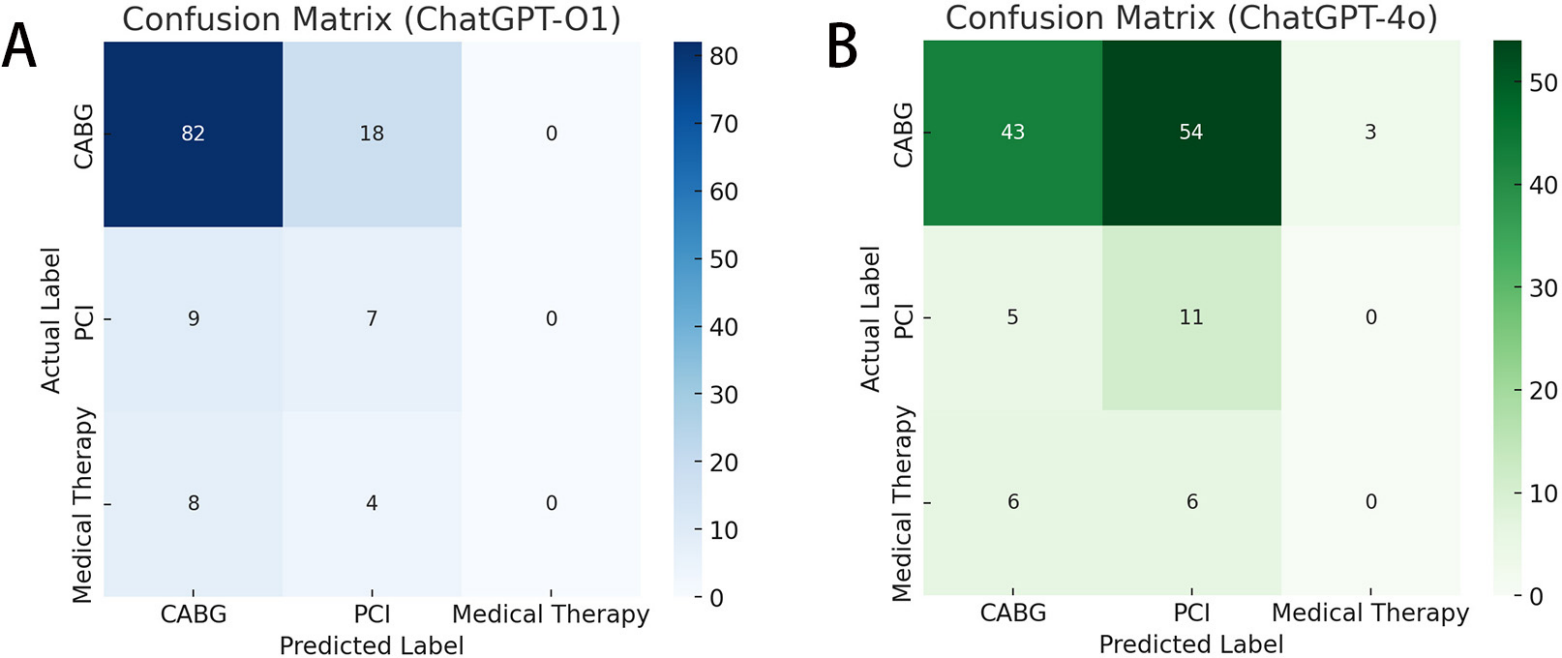
**Comparison of classification performance between the ChatGPT o1 and 4o models**. (A) Confusion Matrix-ChatGPT-O1. (B) Confusion Matrix-ChatGPT-4o.

The two confusion matrices highlight the differences in classification performance between ChatGPT o1 and 4o (Fig. 1A,B). Both models correctly identified most CABG cases (82 vs. 43 true positives), but ChatGPT 4o demonstrated a higher rate of misclassification of CABG cases as PCI (54 cases).

When comparing ChatGPT o1 and 4o (Table 2), ChatGPT o1 showed higher sensitivity and specificity for the CABG cases (82% and 39.3%, respectively) than ChatGPT 4o (43% and 60.7%, respectively). However, ChatGPT 4o demonstrated superior sensitivity for the PCI cases compared with ChatGPT o1 (68.7% vs. 43.7%). Medical therapy was rarely predicted, resulting in zero true positives in both models. The overall accuracy ranged from 46.8% to 88.2% across the cases and models, with F1 scores mirroring this variability. The Cohen’s kappa values were 0.17 (ChatGPT o1) and 0.03 (ChatGPT 4o), indicating low agreement with the multidisciplinary Heart Team’s results. Shannon’s entropy showed greater distributional variability in ChatGPT 4o than in ChatGPT o1 predictions, which suggests more balanced but not necessarily more accurate classification outcomes.

**Table 2.** **Comparison of the performance metrics between ChatGPT o1 and 4o**.

	ChatGPT o1			ChatGPT 4o		
	CABG	PCI	Medical therapy	CABG	PCI	Medical therapy
Sensitivity (%)	82	43.7	0	43	68.7	0
Specificity (%)	39.3	80.3	100	60.7	46.4	97.4
Accuracy (%)	72.6	75.7	90.6	46.8	49.2	88.2
Precision (%)	82.8	24.1	NA	76.9	15.4	NA
F1 score (%)	82.4	31.1	0	55.8	25.3	0
Kappa (95% CI)	0.17 (0.01–0.32)			0.03 (–0.06–0.13)		
Shannon entropy	0.77			1.12		

CABG, Coronary Artery Bypass Grafting; PCI, Percutaneous Coronary Intervention; NA, Not Applicable.

## 6. Discussion

In this study, we evaluated two versions of ChatGPT to determine the alignment of their performances with Heart Team decisions for coronary revascularization and medical therapy in a cohort of 128 patients. Patients with three or more diseased vessels were predominantly assigned to CABG by the Heart Team. Of these patients, 51.6% had three diseased vessels and 37.5% had four or more diseased vessels. Although both models successfully identified a high proportion of CABG cases, ChatGPT o1 showed superior sensitivity (82%) and an F1 score of 82.4% for CABG, indicating a strong performance in recognizing multi-vessel disease that necessitates surgical intervention. By contrast, ChatGPT 4o demonstrated relatively higher sensitivity for PCI (68.7%), indicating that it may be more inclined to recommend percutaneous interventions in borderline cases. Such findings suggest that while LLMs could be explored as a potential tool in the absence of a formal Heart Team, they still demonstrate limitations and errors. However, we believe that with further development, they may play a more integral role in clinical decision-making in the future. Our results are consistent with prior reports that AI-based tools can excel in certain clinical niches while underperforming in others because of inherent model biases and training data constraints [12].

In our center, single-vessel lesions (excluding LAD) and urgent PCI cases are typically not reviewed by the Heart Team. Instead, patients presenting with multi-vessel disease and/or additional comorbidities undergo Heart Team evaluation. In our cohort, 100 (78.1%) of the 128 patients received a CABG recommendation, largely because multi-vessel LAD involvement in non-emergency cases is routinely referred for Heart Team input.

Although both models demonstrated encouraging results for revascularization strategies, particularly CABG, where ChatGPT o1 achieved a sensitivity of 82%, their complete failure to correctly identify candidates for exclusive medical therapy is a critical shortcoming that warrants deeper analysis. Not a single true positive prediction was made for medical treatment, revealing a significant blind spot. This likely reflects the disproportionate emphasis on revascularization cases in the model’s training data [13] but also points to more fundamental limitations in contextual understanding.

Clinical guidelines provide a foundational framework for revascularization decision-making in coronary artery disease. However, in real-world practice, especially when considering CABG, Heart Team decisions are heavily influenced not only by anatomical considerations but also by anticipated postoperative morbidity and mortality risks. These factors include comorbidity burden, vessel quality, pulmonary reserve, and even socioeconomic variables, such as access to postoperative care [14]. Medical therapy decisions are often multifactorial, integrating medical, paramedical, and social determinants that extend beyond the scope of anatomical lesion assessment. The inability of ChatGPT to recommend medical therapy in any case may stem from its limited capacity to account for these nuanced considerations. Unlike expert clinicians, who factor in discharge vulnerability and holistic patient profiles, LLMs likely interpret cases predominantly through the lens of guideline-based revascularization criteria. This mechanistic interpretation fails to capture scenarios in which conservative treatment is preferred due to frailty, high surgical risk, or logistical constraints in postoperative care.

In clinical scenarios, such as breast cancer, bladder cancer, and prostate cancer, treatment approaches and guidelines typically provide clear, stepwise frameworks for clinical decision-making. Previous studies that used ChatGPT in these contexts have demonstrated a high degree of concordance between multidisciplinary team decisions and ChatGPT recommendations, indicating that AI systems can effectively replicate structured, guideline-based clinical decisions [15, 16, 17]. However, in the context of coronary artery disease, particularly complex cases managed by a Heart Team, clinical decision-making frequently involves substantial ambiguity and nuanced judgment calls, creating numerous “gray areas” that are not easily captured by standardized guidelines alone. Consequently, this inherent complexity in cardiovascular decision-making likely contributes to the lower Cohen’s kappa values (0.17 for ChatGPT o1 and 0.03 for ChatGPT 4o) observed in our study. The relatively modest agreement between the ChatGPT recommendations and Heart Team decisions highlights the importance of human expertise and multidimensional clinical judgment, which considers patient-specific factors beyond conventional guidelines. Future development and implementation of AI decision support systems in cardiovascular medicine should aim to integrate more nuanced and patient-centered variables to better align with the complexities encountered in real-world Heart Team discussions [18].

Furthermore, the higher Shannon entropy value observed with ChatGPT 4o (1.12 vs. 0.77 for ChatGPT o1) indicates a more diverse distribution of classifications, but this broader spread did not necessarily translate into higher overall accuracy or better alignment with the multidisciplinary Heart Team. Taken together, these results highlight both the potential and limitations of current LLM-based decision aids in complex clinical pathways, such as cardiac revascularization. Future research should focus on refining input data by incorporating imaging findings, granular risk stratification metrics, and patient-specific factors to reduce misclassification and enhance clinical applicability [19, 20].

The Heart Team concept is not limited to cardiac surgery alone. Similar multidisciplinary teams are used in cancer surgery and transplantation decisions [21, 22]. Recent studies have reported that ChatGPT demonstrates promising performance in supporting clinical decisions [23, 24, 25]. We selected ChatGPT owing to its popularity and extensive database; however, other LLMs such as LLama-2, Olmo, BLOOM, BERT, and Vicuna-13 [26, 27, 28] are also available. As many of these are open-source, researchers can further tailor them to specific clinical or research needs, thereby potentially creating higher-performing models.

### 6.1 Ethics in AI-Human Collaboration

The integration of AI into health-care decision-making raises complex ethical considerations regarding human-AI collaboration, trust, and accountability. Ethical health-care decisions inherently involve nuanced evaluations of patient-specific circumstances, often lacking universally accepted notions of “correct” actions. Therefore, completely delegating ethical medical decisions to AI systems is ethically controversial, highlighting the importance of meaningful human oversight. Health-care settings typically employ collaborative frameworks, such as human-in-the-loop (HITL), where clinicians maintain primary decision authority with AI support, or human-on-the-loop (HOTL), where clinicians supervise and can override AI-generated decisions [29]. Research highlights an intriguing ethical paradox: Clinicians generally trust human counterparts more on moral grounds but often rely heavily on AI owing to its perceived superior technical capability [30]. This paradox may create situations where clinicians, despite moral reservations, trust AI recommendations significantly. Moreover, accountability remains a critical ethical issue. AI systems, perceived as less responsible than human clinicians, shift liability toward developers and providers of these technologies. The minimal impact of autonomy levels (HITL vs. HOTL) on perceived trust and responsibility in health-care contexts suggests that clear human accountability is crucial irrespective of oversight structures. Thus, the ethical deployment of AI in health care must prioritize clearly defined human moral responsibility, emphasizing transparency and accountability to effectively navigate these complex ethical dilemmas.

### 6.2 Shortcomings of ChatGPT

We observed instances in which patients deemed suitable for CABG by the Heart Team were recommended for PCI by ChatGPT. As lesion complexity increases, PCI may be associated with lower survival compared with CABG [31], but the model appeared to overlook such complexity. For example:

#### 6.2.1 Patient 3 (FY, 47-Year-Old Female, EF 55%)

*Findings:* LAD mid 80%, circumflex (CX) 80%, and right coronary artery (RCA) in-stent lesion 90%.

*Model Explanation:* Multiple high-grade stenoses favor PCI as an effective treatment.

#### 6.2.2 Patient 9 (YÇ, 52-Year-Old Male, Hypertension, Obesity, EF 60%)

*Findings:* LAD ostial 90%, LAD mid 100% (retrograde filling via RCA), CX 80%, and RCA proximal 80%.

*Model Explanation:* A critical LAD occlusion with good collateral flow warrants PCI to restore antegrade flow.

In addition, the ChatGPT 4o model incorrectly suggested medical therapy for three patients who did not receive it in actual practice.

#### 6.2.3 Patient 31 (NÜ, 68-Year-Old Female, Valve Pathology, EF 50%)

*Findings:* LAD proximal long-segment 90% stenosis.

*Model Explanation:* “Single-vessel disease with an EF of 50% and no severe dysfunction implies medical therapy is sufficient.”

This reasoning lacks a foundation in the current guidelines or standard clinical practice.

#### 6.2.4 Patient 82 (MMK, 51-Year-Old Male, EF 50%)

*Findings:* LAD proximal 70% stenosis after stent placement.

*Model Explanation:* While the lesion progressed post stenting, it was considered borderline; thus, medical therapy was deemed sufficient.

Again, this conclusion is unsupported by the clinical context provided.

#### 6.2.5 Patient 123 (HHD, 64-Year-Old Male, Hypertension, Diabetes, Obesity, Severe COPD, Chronic Myeloid Leukemia, EF 25%)

**Fig. 2.**
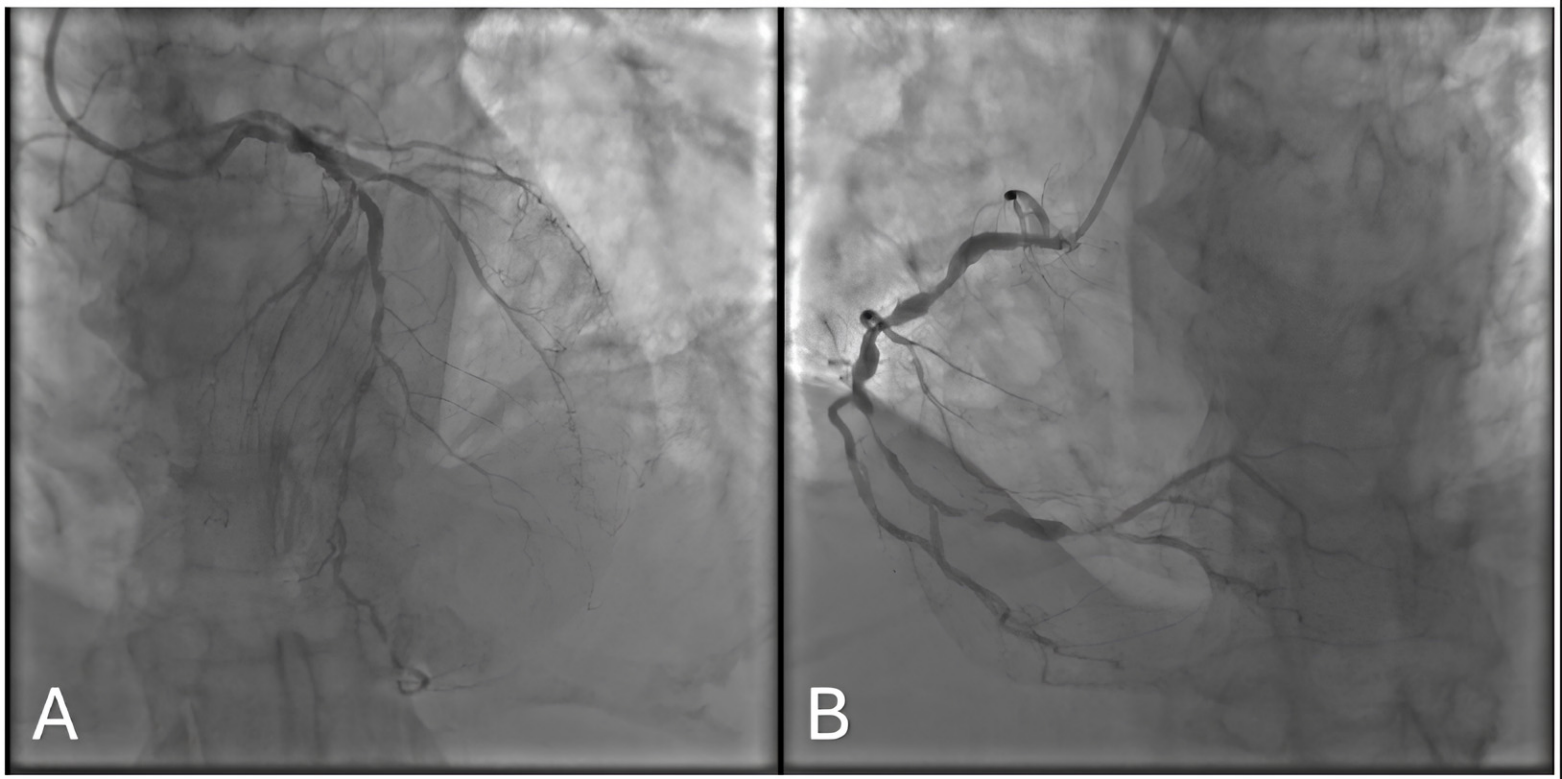
**Coronary angiogram of the 123rd Patient**. (A) Posteroanterior - Cranial 40. (B) Left Anterior Oblique - Caudal 20.

*Findings:* Significant multivessel lesions involving the LAD, CX (Fig. 2A), and RCA (Fig. 2B), with a valvular pathology.

*Rationale:* Owing to the high operative risk and multiple comorbidities, the Heart Team opted for medical therapy.

*Model Explanation:* Both ChatGPT o1 and 4o recommended CABG. Although this might appear reasonable on paper, the patient’s overall status and procedural risks justified a nonsurgical approach.

Conversely, the correct assignment of one patient to maximal medical therapy by the Heart Team underscored the importance of holistic clinical judgment.

Target vessel quality is another critical factor in surgical decision-making. As the LLM in this study processed only numerical data and lesion locations rather than actual angiographic images, it might have overlooked poor target vessels that would compromise grafting success. When angiographic images are not directly analyzed, nuances of lesion location, vessel quality, and surgical feasibility can be lost, which leads to suboptimal or incorrect model recommendations.

#### 6.2.6 Patient 122 (HD, 85-Year-Old Female, Diabetes, EF 35%)

*Findings:* Significant lesions in the LAD, CX, and RCA.

Heart Team Decision: Medical therapy.

*Model Decisions:* ChatGPT o1 recommended PCI, and ChatGPT 4o recommended CABG.

These findings highlight the challenges of using LLMs in real-world clinical decision-making, particularly in complex cardiovascular cases where anatomical details, comorbidities, and surgical risks must be carefully balanced. While ChatGPT shows promise in identifying CABG and PCI candidates, its misclassification of medical therapy cases and its tendency to overlook lesion complexity reveal significant limitations. Without the ability to directly analyze angiographic images or fully grasp patient-specific risk factors, the model struggled to replicate the Heart Team’s nuanced judgment. This suggests that while AI may be a useful decision-support tool, it is not yet ready to replace expert clinical reasoning. Future improvements, such as integrating imaging analysis, refining contextual understanding, and incorporating patient risk stratification, may help bridge this gap and make AI a more reliable companion in complex cardiovascular decision-making.

## 7. Limitations

This study has several limitations. It was conducted retrospectively at a single center with a relatively small sample size, which might have affected the generalizability of the findings. The ChatGPT models did not analyze actual angiographic images and thus could not assess lesion complexity, vessel quality, and surgical feasibility. In addition, the models were not fine-tuned with cardiology-specific data. Only two versions of ChatGPT were evaluated; the results may differ with other language models or prompting methods.

## 8. Conclusion

ChatGPT o1 and 4o offer promising avenues for AI-supported cardiac treatment decision-making, but their utility must be tempered by an understanding of the nuances in model performance. Collaboration between clinicians and data scientists, along with continued model refinement and rigorous validation, will be essential for achieving reliable, context-aware AI systems that can complement, rather than replace, comprehensive Heart Team evaluations.

## Availability of Data and Materials

The datasets used and/or analyzed during the current study are available from the corresponding author on reasonable request.
